# Investigating emotions in Parkinson's disease: what we know and what we still don't know

**DOI:** 10.3389/fpsyg.2013.00336

**Published:** 2013-06-10

**Authors:** Igor Sotgiu, Maria L. Rusconi

**Affiliations:** Department of Human and Social Sciences, University of BergamoBergamo, Italy

**Keywords:** affective tone, emotions, everyday life, experience sampling methods, facial expression of emotion, Parkinson's disease, vocal expression of emotion

## Abstract

Over the last decade, there has been an increasing attention to the role played by emotional processes in Parkinson's disease (PD). However, most of what is known in this area is based on research conducted in laboratory or clinical settings. In this article, the authors underline the need to expand our current knowledge of the psychological correlates of PD by investigating patients' everyday emotions in natural contexts. Specifically, the authors illustrate new research avenues based on the implementation of experience sampling methods. It is argued that these methods could permit future researchers to ecologically assess the frequency and intensity with which parkinsonian patients experience specific emotions (either negative or positive) during their everyday life, providing at the same time precious information on what are the most typical situations in which these emotions occur and on how patients behave in these circumstances. Potential practical implications associated with investigating these issues are discussed.

Parkinson's disease (PD) is a neurodegenerative disorder associated with the loss of dopamine-producing neurons in the pars compacta region of the substantia nigra. The cardinal symptoms of the disease are tremor, muscular rigidity, bradykinesia (i.e., slowness of movement), and postural instability. These motor impairments are often accompanied by a wide range of non-motor symptoms (e.g., depression, executive dysfunctions, concentration difficulty, sleep disturbances, weight loss, autonomic impairments), both symptom categories having a great impact on the quality of PD patient's life (Schrag et al., 2000; Martinez-Martin et al., 2011).

Over the last decade, there has been an increasing attention to the role played by emotional processes in PD. Psychologists and neuroscientists have made important progresses in understanding how PD impairs specific components of emotional processes (e.g., expressive, cognitive, subjective) and have also formulated interesting hypotheses about the underlying neurological mechanisms which could explain the emotional impairments observed in PD patients (for recent reviews, see Blonder and Slevin, 2011; Péron et al., 2012). While the theoretical and empirical work done so far has been very precious, we believe that the research on the emotional aspects of PD is only at the beginning and researchers have the opportunity to gain more information on this topic.

The main aim of the present article is to delineate unexplored research directions for investigators studying the emotional aspects of PD. In the following, we first briefly summarize what researchers already know about emotional processes affected by PD. Then, we critically discuss the most important limitations of the current empirical literature and present a potential new research area which could advance our understanding of the emotional processes in PD.

## Current research areas

Researchers investigating emotions in PD have devoted much of their efforts in three major areas: (1) Facial and vocal expression of emotion, (2) recognition of emotional stimuli, (3) changes in PD patients' affective tone and mood. Let's consider the main results obtained in each area.

### Facial and vocal expression of emotion

Researchers working within this area designed well-controlled laboratory studies in order to investigate how PD patients express emotions in the face and voice. Overall, it has been found that the expression abilities of PD patients are significantly compromised. For example, Simons et al. (2003) assessed the facial activity of 22 PD patients and 22 healthy control participants using the Facial Action Coding System (FACS; Ekman and Friesen, 1978). Results showed that PD patients exhibited reduced levels of spontaneous and voluntary expressions in response to unpleasant olfactory stimuli as compared with controls. Similarly, Möbes et al. (2008) employed a software for speech analysis (PRAAT; Boersma and Weenink, 1996) to assess the productions of emotional prosody in 16 PD patients (with no signs of dysarthria) and 16 controls, both groups being matched for age, gender, and education. More in detail, subjects participating in this study were asked to produce the name “Anna” using a specific emotional intonation (i.e., happy, sad, or neutral). Findings indicated that patients with PD showed smaller pitch and loudness ranges than controls, thus performing worse in this task; however, the two groups did not significantly differ in two supplementary tasks assessing their phonation capacity and their ability to imitate a professional speaker. On the basis of these results, Möbes et al. concluded that motor impairment is not a sufficient condition to explain changes of emotional speech in PD patients.

### Recognition of emotional stimuli

The investigation of emotion recognition skills in parkinsonian patients is probably the most representative research area within the emotion literature on PD. Indeed, a huge number of studies have been conducted in the last years with the goal to understand if PD patients dealing with different disease stages are still able to correctly identify, discriminate, and rate the emotional content of stimuli (e.g., pictures, prerecorded speech samples, written sentences) presented in laboratory settings. Unfortunately, the data collected so far are inconsistent and quite difficult to interpret: whereas some researchers reported that PD patients perform worse than controls in a number of recognition tasks, there is also evidence that the two groups do not significantly differ in the same tasks (for reviews, see Assogna et al., 2008; Schröder et al., 2010; Péron et al., 2012). A recent meta-analysis by Gray and Tickle-Degnen (2010) has shed some lights on this controversial point. These authors examined the magnitude of the association between PD and deficits in emotion recognition using data from 34 independent comparisons, which involved a total of 1295 participants (594 individuals with PD and 701 controls). It emerged that about 75% of the comparisons evidenced a significantly poorer performance among the parkinsonian patients relative to the controls. When looking at the factors moderating the participants' emotion recognition abilities, Gray and Tickle-Degnen found that PD groups were more impaired in recognizing emotion from prosodic stimuli than from facial displays. Moreover, it was found that the performances of PD patients were poorer when the stimuli to be recognized referred to negative emotions (e.g., anger, disgust, sadness) as compared to positive ones (e.g., happiness).

### Changes in affective tone and mood

This research area concerns the complex changes in affective tone and mood of PD patients. Recent prevalence studies (e.g., Aarsland et al., 2007; Kulisevsky et al., 2008; Nègre-Pagès et al., 2010; Siri et al., 2010) have documented that patients with PD frequently report or exhibit signs of anxiety, apathy, and depressed mood. These emotional dysfunctions may affect up to 70% of PD patients, in some cases taking the form of psychopathological syndromes with a clinical significance and requiring pharmacological and psychological treatment. Importantly, it has been suggested that some patients may experience significant affective and mood changes as a result of surgical treatments of PD. In particular, depression and anxiety have been reported following deep brain stimulation of the subthalamic nucleus (e.g., Berney et al., 2002; Houeto et al., 2002; Perriol et al., 2006). However, these complications seem more likely in patients with preoperative emotional dysfunctions (for reviews, see Temel et al., 2006; Bronstein et al., 2011).

Although there are PD patients exhibiting very specific emotional dysfunctions, it has been demonstrated that a significant proportion of parkinsonian individuals receive comorbid diagnoses, with depression-apathy and depression-anxiety co-occurring most frequently (on this topic, see for example Dissanayaka et al., 2010; Oguru et al., 2010). Interestingly, in a study conducted on a group of 58 PD patients without dementia, Costa et al. (2006) found that individuals with high levels of depression were more likely to display features of alexithymia (i.e., alteration of the ability to describe and identify one's feelings, and to distinguish feelings from bodily sensations of emotional arousal; cf. Taylor et al., 1991). However, more recent investigations comparing PD patients and control subjects (Costa et al., 2010; Assogna et al., 2012) have suggested that depression and alexithymia might be independent disorders in PD. There is also empirical evidence that depressive symptoms may precede the onset of PD. For example, Leentjens et al. (2003) used data from a computerized practice-based register to calculate the incidence of depression in a group of 338 Dutch citizens later diagnosed with PD. Results showed that, at the time of diagnosis of PD, 9.2% of the PD patients had a history of depression relative to 4.0% of a matched control population; the difference in incidence was statistically significant. These findings, along with other ones reported in epidemiological studies conducted with similar methods (e.g., Nilsson et al., 2002; Fang et al., 2010), suggest that depressed mood may constitute either a symptom or a risk factor for PD (cf. Blonder and Slevin, 2011).

In concluding this section, we would like to remark that while the here used concepts of *affective tone* and *mood* are not exactly equivalent to the one of *emotion*, all these concepts refer to strongly interconnected phenomena, as prolonged emotions tend to transform into mood-like and affective states (see, Plutchik, 1994). Furthermore, the self-report instruments which are commonly used to assess mood and affective alterations in PD populations—for example, the Beck Depression Inventory (Beck et al., 1996) or the Spielberger State-Trait Anxiety Inventory (Spielberger et al., 1983)—explicitly refer to the patients' everyday emotions such as worry, sadness, guilt, and interest.

## Future directions: studying the everyday emotional life of PD patients using experience sampling methods

Altogether, the studies reviewed in the previous section clearly show that PD negatively influences the affective life of patients suffering from this disorder. More in detail, research on emotional expressions suggests that PD patients may exhibit significant deficits in the non-verbal communication, having difficulties in producing both emotional facial movements and affective prosody. Moreover, it has been demonstrated that patients with PD may display deficits in performing emotion recognition tasks in laboratory settings: this seems particularly true for experimental tasks involving prosodic stimuli with a negative valence. Lastly, a great number of studies have shown that PD patients frequently experience mood alterations and emotional dysfunctions, including depression, apathy, anxiety, as well as alexithymia.

From what has been said so far, it appears that investigating the emotional consequences of PD may shed light on the complex relationship between the neurobiological and the psychological aspects of the disease. However, a closer examination of the available literature reveals that there are some relevant issues which have not yet been explored. With this regard, we note that although researchers have documented that PD is associated with significant changes in the overall affective tone and mood of patients, to the best of our knowledge there have been no studies aimed at investigating the quality of their everyday emotional life. In our opinion, there is urgency to understand how parkinsonian individuals psychologically adapt to their disease and emotionally respond to the diverse stimuli which characterize their everyday life environment. Indeed, it is undeniable that both motor and non-motor symptoms, as well as the therapeutic interventions aimed at reducing their severity, dramatically influence the emotional life of people suffering from PD. Based on these considerations, we believe that it would be important to empirically determine the frequency and the intensity with which PD patients experience specific categories of emotions—either negative (e.g. fear, sadness, anger, disgust) or positive (e.g., joy, contentment, relief, hope)—in their daily life. We also argue that researchers would greatly benefit from knowing what are the most typical situations in which PD patients experience negative and positive emotions, how individuals with PD behave in these situations, and how everyday emotional experiences might affect the physical and psychological well-being of patients over time. From a methodological point of view, we propose the introduction of research methods able to assess the content of patients' emotional experiences and the contextual factors associated with these experiences. In particular, a promising approach could be the use of *experience sampling methods* (for an overview, see Bolger et al., 2003; Mehl and Conner, 2011). Hereafter, we describe what these methods consist of and how they could be usefully applied with the goal to investigate the PD patients' everyday emotional life.

Different from other research methodologies frequently employed in the study of everyday emotional life (e.g., retrospective questionnaires; see Scherer et al., 2004), the experience sampling methods allow researchers to assess people's emotional experiences *on line* (i.e., while they are actually happening) or shortly after (some minutes or hours) from their occurrence. More in detail, researchers following this approach ask participants to record events they have personally experienced during their everyday life, answering written questions concerning both their emotional reactions to what happened and the specific situational context in which the recorded events occurred. Generally, this information is gathered by means of paper-and-pencil diaries or booklets (Zelenski and Larsen, 2000; Brandstätter, 2001; Vansteelandt et al., 2005; Nezlek et al., 2008). However, recent studies—typically conducted in non-clinical samples (e.g., Pe and Kuppens, 2012)—have introduced palmtop computers, serving the same functions of paper-and-pencil self-report instruments (on this topic, see Barrett and Barrett, 2001; Le et al., 2006). Importantly, researchers using experience sampling methods have to carefully choose an appropriate sampling protocol that is, they have to establish the criteria according to which participants will select and report their experiences (e.g., closely following a target event, at fixed times throughout the day, in response to a random signal). Furthermore, decisions must be taken on the length of the whole sampling period, namely how long the participants will have to record their experiences (e.g., 1 day, 3 days, 1 week, 1 month). When taking all these decisions, researchers should pay special attention to the specific objectives of their experience sampling study. Other important factors to be taken into account include the burden to participants and the prevalence of the target events and psychological states under investigation (for a detailed discussion, see Reis and Gable, 2000; Conner Christensen et al., 2003).

Although it is undeniable that experience sampling methods may pose significant obstacles to both researchers and study participants (on this issue, see Scollon et al., 2003), we argue that the application of this approach to parkinsonian populations could bring significant advantages. The most important benefit is the ecological validity of the studies implementing experience sampling methods. As we have noted in the first part of this article, our current knowledge of the emotional aspects of PD mainly comes from studies conducted in laboratory and clinical settings. However, since these contexts significantly differ from real life, the generalizability of findings obtained from this research is limited. Contrary to this, experience sampling methods allow researchers to assess emotional processes and affective states occurring in natural settings, thus permitting greater generalizability of their results. A second advantage associated with the use of experience sampling methods in PD research concerns the possibility to investigate the contingencies between the emotional experiences occurring in the life of PD patients and specific disease-related factors such as, for instance, taking dopamine agonist drugs, dealing with motor fluctuations, performing a rehabilitation activity, and so on. Finally, it is well-known that experience sampling methods permit to reduce memory fallacies, social desirability tendencies, and other biased response patterns which are typically related to the use of traditional self-report measures (cf. Bolger et al., 2003; Scollon et al., 2003). With this respect, two experimental studies by Smith et al. (2006) employed survey methods to investigate how PD patients assess their satisfaction with life and health. It was found that judgments formulated by the participants significantly varied as a function of how experimenters introduced the study. In our opinion, these results strongly suggest to consider with caution data on PD patients' quality of life collected in the context of survey research.

In the light of the abovementioned advantages, we argue that researchers should use experience sampling methods to investigate the everyday emotional life of parkinsonian patients who are facing different clinical conditions: for instance, patients newly diagnosed with PD who have never received pharmacological treatment (they are typically called *de novo patients*); patients taking dopamine agonist medications and exhibiting frequent and intense motor fluctuations; pharmacologically treated patients showing both motor and non-motor fluctuations; patients who do not exhibit disease-related symptoms because they are positively responding to the treatment with antiparkinsonian drugs; patients submitted to deep brain stimulation surgery. We believe that the comparison between these patient categories might allow researchers to better describe how the emotional life of parkinsonian patients changes in the various disease stages, providing at the same time precious information about the contextual factors which are able to affect these transformations. From a methodological point of view, these research aims could be best attained implementing both cross-sectional and longitudinal designs. Specifically, longitudinal studies could offer the researchers the opportunity to analyze changes in the everyday emotional life of PD patients at the within-person level. This would also permit to better understand the efficacy of pharmacological and surgical treatments over time and, more generally, the progression of disease.

Of course, researchers conducting experience sampling studies in parkinsonian groups should carefully adapt their methodology to the specific characteristics of the study participants they are investigating. For instance, if it has been planned to conduct a study of pharmacologically treated patients with severe motor fluctuations, it would be recommendable to give the participants the opportunity to report their emotional experiences shortly after their “off” phases (cf. Hughes et al., 1994), thus reducing the burden of research assessments. Importantly, researchers should also evaluate how to best collect patients' self-reports: for instance, while palmtop computers may be unsuitable for older patients, these instruments could be fruitfully implemented in studies of younger patients. Lastly, great attention should be paid in defining experience sampling protocols that are appropriate with respect to the patients' disease stage.

## Conclusion

To conclude, we note that investigating the everyday emotional life of PD patients could advance our understanding of the psychological aspects of PD, providing at the same time precious indications on how to support people suffering from this disorder. Indeed, knowing in which situations and circumstances PD patients experience negative emotions such as anger, sadness, anxiety, and shame, could shed insight into the main difficulties and obstacles these individuals face in their everyday life. On the other hand, knowing in which situations and circumstances PD patients experience positive emotions such as joy, contentment, serenity, and interest, could facilitate the identification of the contextual factors which are presumably associated with higher levels of quality of life and subjective well-being. At a practical level, this knowledge may be used to design and implement psychological interventions targeting both the PD patients and their caregivers. Such interventions should be aimed at promoting an improved ability to read and interpret the meaning of the emotions that occur in everyday life. This is another way to help patients and their caregivers to use emotions as valuable tools through which they could not only identify the risks and threats of their environment, but also discover and get in touch with its resources and opportunities.

### Conflict of interest statement

The authors declare that the research was conducted in the absence of any commercial or financial relationships that could be construed as a potential conflict of interest.
